# No sex-specific difference in disease trajectory in multiple sclerosis patients before and after age 50

**DOI:** 10.1186/1471-2377-13-73

**Published:** 2013-07-03

**Authors:** Riley Bove, Alexander Musallam, Brian C Healy, Maria Houtchens, Bonnie I Glanz, Samia Khoury, Charles R Guttmann, Philip L De Jager, Tanuja Chitnis

**Affiliations:** 1 Partners Multiple Sclerosis Center, Department of Neurology, Brigham and Women’s Hospital, Brookline 02445, MA, USA; 2 Harvard Medical School, Boston 02115, MA, USA; 3 Massachusetts General Hospital Biostatistics Center, Boston 02114, MA, USA; 4 Center for Neurologic Disease, Harvard Medical School, 77 Avenue Louis Pasteur, NRB168, Boston 02115, MA, USA

**Keywords:** Demyelinating disease, Multiple sclerosis, Natural history studies, MRI, Disease progression, Menopause, Gender

## Abstract

**Background:**

The disease course in multiple sclerosis (MS) is influenced by many factors, including age, sex, and sex hormones. Little is known about sex-specific changes in disease course around age 50, which may represent a key biological transition period for reproductive aging.

**Methods:**

Male and female subjects with no prior chemotherapy exposure were selected from a prospective MS cohort to form groups representing the years before (38–46 years, N=351) and after (54–62 years, N=200)age 50. Primary analysis assessed for interaction between effects of sex and age on clinical (Expanded Disability Status Scale, EDSS; relapse rate) and radiologic (T2 lesion volume, T2LV; brain parenchymal fraction, BPF) outcomes. Secondarily, we explored patient-reported outcomes (PROs).

**Results:**

As expected, there were age- and sex- related changes with male and older cohorts showing worse disease severity (EDSS), brain atrophy (BPF), and more progressive course.

There was no interaction between age and sex on cross-sectional adjusted clinical (EDSS, relapse rate) or radiologic (BPF, T2LV) measures, or on 2-year trajectories of decline.

There was a significant interaction between age and sex for a physical functioning PRO (SF-36): the older female cohort reported lower physical functioning than men (p=0.002). There were no differences in depression (Center for Epidemiological Study – Depression, CES-D) or fatigue (Modified Fatigue Impact Scale, MFIS) scores.

**Conclusions:**

There was no interaction between age and sex suggestive of an effect of reproductive aging on clinical or radiologic progression. Prospective analyses across the menopausal transition are needed.

## Background

Multiple sclerosis (MS) affects almost three times more women than men. The incidence and disease course of MS are modulated by a complex interplay among genetic susceptibility, sex hormones, and the environment [1-3]. Both sex and age influence disease course, with both advanced age and male sex associated with worsening disability and more rapid progression of disease [4-8]. Interestingly, we found that women whose onset of MS symptoms occurred after age 50 are more likely to have a progressive course than women with an earlier symptom onset, and the female: male ratio for individuals in this group is lower than in individuals with onset between ages 18-49 [9].This suggests that the endocrine changes associated with menopause may reduce sexual dimorphism in disease course in later years.

In this study, we use prospectively collected data to examine disease course in men and women with MS in cohorts that represent the years before and after age 50. We examine the interaction between age and sex to test the hypothesis that women experience acceleration in their trajectory of decline after age 50, relative to similarly aged men [10].

## Methods

### Subjects

The Partners Multiple Sclerosis Center is a regional MS referral center in the Northeastern United States. To date, over 2000 of our patients are enrolled in our detailed longitudinal cohort study (Comprehensive Longitudinal Investigation of Multiple Sclerosis at the Brigham and Women’s Hospital, CLIMB), with follow-up for an average of 2.6 (SD 2.8) years. All CLIMB patients have a diagnosis of MS as defined by the 2005 McDonald criteria, or a clinically isolated syndrome (CIS) with an MRI suggestive of MS. Demographic characteristics (race and ethnicity by NIH criteria) and complete MS history are recorded upon enrollment. Neurological examinations are obtained every 6 months, and brain MRIs annually. We excluded subjects with prior exposure to cyclophosphamide and mitoxantrone, to minimize potential confounding effects of iatrogenic menopause.

For this study, we divided subjects into twoage cohorts, aged before and after 50: Cohort 1 (C1): ages 38–46 (n=351), and Cohort 2 (C2): ages 54–62 (n=200). The menopausal transition involves a series of physiological changes associated with reproductive senescence in women, divided into a series of stages [11]. Operationally, menopause is defined as the final menstrual period, after which no further menses occur during a 12-month interval.The mean age at menopause in Western societies is 49-52 [12,13], and preliminary analysis of a reproductive history questionnaire in our clinic cohort suggests a median age of 50.7 years (unpublished data). Thus, while recognizing variability in individual age at menopause, cohorts C1 and C2 were designed to capture women before and after age 50 (ages 38–46 and 54-62), to minimize the effect of marked hormonal fluctuations at the time of menopause itself on disease characteristics.A subset of these subjects had at least two years of follow-up and therefore contributed to our analysis of change in the prior two years (N=169 and 97). We included men in these age groups in addition to women in order to provide a control group for sexcomparisons. Institutional Review Board approval was granted by the Partners Human Research Committee. Informed consent for use of clinical data and MRI information was obtained from all study subjects.

### Clinical outcomes

The expanded disability status scale (EDSS [14]) score and the disease category (CIS, relapsing remitting, secondary progressive, primary progressive [15]) were recorded every 6 months by the treating physician. In addition, the occurrence of clinical relapses in the interval between visits was recorded.

### MRI protocol/segmentation

Subjects were scanned with a T2-weighted axial dual echo protocol covering the whole brain (TR=3000 ms, TE=30/80 ms, 192 phase encoding steps, 256×256×54 voxels with 0.93 × 0.93×3 mm^3^ nominal voxel size and no interslice gaps). All baseline and follow-up scans were performed on the same 1.5 Tesla machine (GE Signa, General Electrics, Milwaukee, WI).T2LV was determined by semi-automated outlining using local thresholding and manual editing (3D Slicer 3.4, http://www.slicer.org/). Automated template driven segmentation identified normal appearing white matter, grey matter and CSF. Head size normalized BPF was calculated as follows: (grey matter + white matter + T2LV)/intracranial volume [16].

### Patient reported outcomes (PROs)

In a subgroup, the following scales were completed annually: Center for Epidemiological Studies Depression Scale (CES-D [17]), Modified Fatigue Impact Scale (MFIS [18]) for fatigue, and general quality of life (SF-36 [19]). The MFIS and SF-36 were scored using the algorithm from the Multiple Sclerosis Quality of Life Inventory [20].

### Statistical methods

Our primary goal was to assess the interaction between age and sex on each of our clinical and MRI measures. Secondarily, we assessed PROs.

The patient characteristics at the last visit were used for this first set of analyses. To determine if the impact of age was different in the two sexes, the interaction between age group and sex was assessed using linear regression for the MRI and PRO outcomes, negative binomial regression for the number of attacks in the previous 2 years, and proportional odds logistic regression for EDSS. The T2 lesion volume was log-transformed for this analysis. An analysis using the cube root of the lesion volume produced the same results as the analysis using the log-transformed lesion volume, and thus only the results from the latter are presented. The interaction analysis was also completed adjusting for disease duration and disease category. There were two models for disease category; the first included an indicator for current relapsing course (CIS, RRMS) versus current progressive course (PPMS, SPMS), while the second included an indicator for relapsing onset (CIS, RRMS, SPMS) versus progressive onset (PPMS). Since the results were largely unchanged between these two analyses, only the first are reported. This analysis compared the C1 and C2 cohorts. In addition to the interaction analysis, we investigated the separate effect of age and sex by stratifying on both sex and age. For our investigation of the effect of age, the two cohorts were compared in the males and females separately using a t-test for PROs, age, age at onset, and disease duration; a Wilcoxon test for the EDSS and relapse rate; and Fisher’s exact test for distribution of white, Hispanic and disease type. To investigate the impact of sex with each age cohort, the same approaches were used.

In addition to the characteristics at last visit, the rate of change in the EDSS, MRI and PRO outcomes were investigated using a random intercepts model. As in the cross-sectional analysis, the primary analysis investigated the interaction between age and sex on the rate of change. The secondary analyses compared the rates of change in the age cohorts for each sex separately, and the rate in each sex was compared in each cohort separately. All analyses were completed in the statistical package R (http://www.r-project.org) and SAS Software (Version 9.2).

## Results

### Patient characteristics

We found that increasing age affected the distribution of several clinical parameters among CLIMB patients from the Partners MS Center (Boston, MA) who were from cohorts aged under 50 (C1: 38–46 years) and over 50 (C2: 54–62 years) (Table 1). Most notably, in both men and women, individuals in C2 had an older age at onset (p<0.0001). Nonetheless, the mean disease duration from symptom onset remained longer in the older C2 subjects (women p<0.0001 and men p=0.02). While the proportion of RRMS/CIS subjects declined in the older cohort of subject, this parameter was only significantly different in women (p<0.0001).

**Table 1:** Sociodemographic characteristics presented by sex and age cohort (p-values for comparison analyses are provided)

	**Female**			**Male**			**Male vs. Female**	
**Cohort (years)**	**C1**	**C2**	**C1 vs. C2**	**C1**	**C2**	**C1 vs. C2**	**C1**	**C2**
	**38-46**	**54-62**	**p**	**38-46**	**54-62**	**p**	**p**	**p**
**N**	258	153		93	47			
**Age (yrs)**							0.32	0.29
**mean**	42.36	57.97		42.04	57.51			
**std. dev**	2.66	2.47		2.56	2.66			
**Race (% white)**^**+**^	95.2	95.4	1	95.7	93.6	0.687	1	0.703
Ethnicity (% hispanic)*	3.6	0.7	0.098	1.1	0	1	0.301	1
**Current course type (%)**			<0.0001			0.0031	0.0011	0.0097
Relapsing (RRMS, CIS)	96.5	77		86	72.3			
SPMS	2.4	15.7		10.8	6.4			
PPMS	1.2	6.5		3.2	21.3		1.2	6.5
**Disease duration (yrs)**			<0.0001			0.01	0.71	0.01
**Mean**	9.93	16.76		9.68	13.15			
**std. dev.**	6.02	9.47		5.66	8.45			
**Age at disease onset (yrs)**	32.42 (6.27)	41.21 (9.65)	<0.0001	32.37 (5.99)	44.36 (9.24)	<0.0001	0.94	0.05
RRMS, CIS Mean (SD)	32.39 (6.33)	41.32 (9.76)	<0.0001	32.94 (5.72)	44.35 (8.75)	<.00001	0.49	0.105
SPMS Mean (SD)	31.83 (4.17)	37.71 (8.65)	0.120	27.90 (5.78)	38.67(10.41)	0.036	0.169	0.860
PPMS Mean (SD)	36.00 (4.36)	46.20 (8.18)	0.067	32.00 (9.54)	46.10 (10.87)	0.069	0.545	0.982

^+^8 missing and did not contribute to the analyses *9 missing and did not contribute to the analysis.

When we compared men and women within each age cohort, significantly more women than men had a relapsing course (p=0.001) in C1. In C2, women had a modest younger age at symptom onset (p=0.05) and increased disease duration (p=0.01).

### Objective clinical and radiologic outcomes

### Cross-sectional analysis

To address the question of whether disease course changes after age 50, we assessed whether there was evidence for statistical interaction between age and sex in their influence on clinical (EDSS, relapse rate) and radiologic (T2 lesion volume, BPF) outcomes when comparing the C1 and C2 age cohorts. No evidence for interaction was observed in the unadjusted analysis and in analyses adjusted for disease duration and disease category (Table 2). Thus, the difference between women and men was similar in both age cohorts on cross-sectional analysis.

**Table 2:** **Cross**-**sectional disease characteristics compared by age cohort** (**ages 38**–**46 vs**. **54**–**62 years**) **and by sex**

	**Female**			**Male**			**Female vs. Male**		Adjusted interaction analysis age cohort*sex
**Cohort (years)**	**C1**	**C2**	**p**	**C1**	**C2**	**p**	**C1**	**C2**	
	**38**-**46**	**54**-**62**		**38**-**46**	**54**-**62**		**p**	**p**	
**Clinical characteristics (mean +/- SD)**									
EDSS*	1.42 (1.33)	2.39 (1.99)	<0.0001	1.98 (1.88)	2.82 (2.31)	0.02	0.03	0.32	0.6
**Relapse rate in prior two years %**	0.34 (0.64)	0.05 (0.27)	<0.0001	0.41 (0.83)	0.05 (0.22)	0.03	0.84	0.84	0.84
**MRI characteristics (mean +/- SD) +**									
**BPF**	0.88 (0.04)	0.85 (0.04)	<0.0001	0.85 (0.04)	0.81 (0.04)	<0.0001	0.0002	0.0004	0.34
**T2 Lesion volume**	3.91 (3.06)	5.29 (5.76)	0.11	4.42 (3.84)	6.11 (6.31)	0.41	0.36	0.63	0.98
**Patient-reported outcomes** (**mean +/- SD) ^**									
**CES-D**	29.5 (8.28)	28.18 (8.24)	0.26	30.94 (8.22)	29.86 (8.53)	0.63	0.3	0.43	0.93
**MFIS**	24.64 (16.27)	28.52 (16.9)	0.1	27.33 (20.23)	28.24 (19.52)	0.86	0.42	0.95	0.5
**SF**-**36 *PCS***	49.08 (8.99)	45.34 (11.08)	0.01	48.85 (9.14)	46.44 (10.77)	0.37	0.88	0.68	0.55
**SF**-**36 *MCS***	49.05 (10.02)	50.03 (9.26)	0.48	47.51 (9.12)	48.11 (8.7)	0.8	0.33	0.37	1

Legend: *EDSS*, expanded disability status scale, *BPF*, brain parenchymal fraction, *CES-D*, Center for Epidemiological Study, *MFIS*, Modified Fatigue Impact Scale *: N=551. The group comparisons were completed with a Wilcoxon test, and the interaction analysis was completed with a proportional odds model %: N=285. The group comparisons were completed with a Wilcoxon test, and the interaction analysis was completed with a negative binomial regression model +: N=308. The BPF and log-transformed T2 lesion volume were compared using a t-test, and the interaction analysis was completed with linear regression ^: N=287. All comparisons were made using a t-test, and the interaction analysis was completed with linear regression.

Comparing C1 and C2, we saw the expected increase in EDSS and decline in relapse rate and BPF with increasing age, for both women (p<0.0001) and men (p=0.02 for EDSS, p=0.03 for relapse rate and p<0.0001 for BPF) (Table 2). When we compared women and men in each age cohort, C1 women had a lower EDSS than men (p=0.03).Additionally, BPF was consistently lower in men relative to women in both age cohorts (p<0.001)

### Two-year longitudinal analysis

Given the availability of longitudinal data, we also examined the accumulation of MS-related disability in subjects aged before and after 50 years. Specifically, we compared the slope of change over a 2-year period in clinical and radiologic parameters between cohorts C1 and C2. The interaction analysis demonstrated no significant interaction between age and sex in any outcome (Table 3).

**Table 3:** **Longitudinal change in clinical**, **radiologic and patient**-**reported outcomes compared by age cohort** (**ages 38**–**46 vs**. **54**–**62 years**) **and by sex** (s**lopes of change over preceding 2 years are compared)**

	**Female**			**Male**			**Female vs. Male**		**Adjusted interaction analysis**
**Cohort (years)**	**C1**	**C2**	**p**	**C1**	**C2**	**p**	**C1**	**C2**	
	**38-46**	**54-62**		**38-46**	**54-62**		**p**	**p**	Age Cohort*Sex
**N**	**128**	**78**		**41**	**19**				
**Clinical characteristics**									
**EDSS**	0.139	0.122	0.83	0.152	-0.066	0.11	0.876	0.201	0.22
**MRI characteristics**									
**BPF**	-0.001	-0.001	0.7	-0.002	-0.004	0.3	0.336	0.193	0.48
**T2 lesion volume**	-0.002	0.025	0.34	0.019	-0.038	0.18	0.54	0.121	0.14
**Patient-reported outcomes**									
**CES-D**	0.22	0.705	0.36	-0.146	0.056	0.82	0.526	0.456	0.74
**MFIS**	0.17	1.045	0.27	-0.475	0.806	0.37	0.549	0.846	0.86
**SF-36 *PCS***	-0.061	-0.349	0.5	0.196	1.111	0.26	0.628	0.052	0.16
**SF-36 *MCS***	-0.357	-1.304	0.17	0.259	-0.858	0.33	0.419	0.632	0.99

Legend: *EDSS*, expanded disability status scale, *BPF*, brain parenchymal fraction, *CES-D*, Center for Epidemiological Study, *MFIS*, Modified Fatigue Impact Scale.

Additionally, the slope of decline in these clinical and MRI parameters was not significantly different between the C1 and C2 age cohorts, suggesting that, within the sampled time frame, there does not appear to be a significant acceleration of decline as MS subjects go from earlier to later middle age. Men and women also did not differ in either cohort for 2-year changes in clinical and radiologic parameters.

### Patient reported outcomes (PRO’s)

To explore the possibility that other features of MS may be influenced by reproductive aging, we assessed cross-sectional and longitudinal data collected using three different instruments: CES-D (depression), MFIS (fatigue) and SF-36 (quality of life, QOL). In the cross-sectional summary measures for these instruments, our analysis revealed a significantly lower physical symptom summary score (PCS) in the C2 female cohort (p=0.01) relative to C1 women, but not in the male cohorts (p=0.88) (Table 2). In a secondary analysis of the SF-36 sub-scores, we noted the presence of a substantially lower SF-36 “physical functioning” score for women in C2 relative to C1 (p<0.0001), but not for men (p=0.46) (Additional file 1: Table S1). However, there was no interaction between age and sex and any PRO outcome, in either the unadjusted or the adjusted analyses.

In longitudinal analysis, the secondary analysis of the SF-36 sub scores revealed a significant interaction between age and sex in terms of the change in the overtime in the physical functioning sub score (p=0.002). There were no other significant differences between female age cohorts after a Bonferroni correction for multiple comparisons (Additional file 1: Table S1). There were no other significant interaction terms in longitudinal analyses of our PRO data. Thus, while there was no acceleration noted in the trajectories of decline of subjects after the age of 50 using clinical and MRI measures, female subjects may report an altered perception of their physical function at this stage of their life course.

## Discussion and conclusion

In this study, we investigated whether female subjects may experience a more rapid decline after age 50, out of proportion with the age-related decline that male subjects also experience at this stage of their life. Estrogen may mediate neuroinflammatory signals and protect against neurodegeneration [21-23]. Consistent with prior studies, we found that both older age and male sex influence the disease course of MS, with men displaying a more progressive disease and more rapid accumulation of disability as measured by clinical and radiological outcomes [24,25]. However, we did not find any interaction between age and sex that would suggest a differential effect of menopause on these outcomes.

In other inflammatory disorders, such as systemic lupus erythematosis and rheumatoid arthritis, the onset of menopause has been associated with altered disease trajectory [26], presumably through estrogenic modulation of immune cells. In epilepsy, another neurologic disorder, the perimenopause is associated with exacerbation of catamenial seizures, and the menopause, with a decrease in seizure frequency. This may occur because of changes in estrogenic modulation of synaptic regulation. Peri-menopausal hormone replacement therapy (HRT) has been associated with modulatory effects in these other neurologic [27] and inflammatory diseases [26]. To our knowledge there are no clinical data specifically assessing an association between menopause and disease trajectory in multiple sclerosis, and prior patient reports have been variable, with a subset of women reporting worsening of symptoms with menopause [28-30]. These small studies also differed vastly in the reported effectiveness of HRT, from limited utility to improvement in 75% patients.

Given our prior analyses revealing that MS with onset after age 50 (late-onset MS) is more similar in women and men than MS with onset before age 50, suggesting an effect of menopause [9], the findings in this study were somewhat surprising. While an expected age-related decline was observed, it was seen both in men and women, suggesting that the change is not driven primarily by changes in female sex hormones.

Patient-reported outcomes may capture different aspects of subjects’ experience with MS than episodic clinical and radiological assessments [31,32]. The findings of worsening physical functioning in older cohort of women suggest some sex-specific acceleration of functional decline or at least in the perception of this decline.

The major limitation of this study is that we were presently unable to assess the specific age at menopause in our female cohorts. Instead, we compared the trajectories of men and women in defined age cohorts using an approximation of the mean age of menopause in Western society, and excluding individuals with exposure to chemotherapy, rather than following a cohort of women through individual, well-characterized menopausal transition. During the menopausal transition, endocrine shifts may lead to a greater variability in inflammatory activity, whereas thereafter, much lower estrogen levels may make male and female profiles more similar and may be associated with more rapid clinical deterioration. Thus, long-term longitudinal analyses of women throughout their menopausal transition may be more informative about specific endocrine-associated disease patterns; however we were not able to address this question with our current dataset. Second, given the exploratory nature of this study, we included all available patients in each age cohort, leading to limited older males in particular. Given our sample sizes (258 females in C1, 153 females in C2, 93 males in C1 and 47 males in C2), we had 80% power to detect interaction effects of at least 0.575 standard deviations for any of the measures. Since this is a moderate effect size, our results demonstrate that the difference between men and women in the two age groups is not large, but to detect a mild to moderate effect size larger sample sizes would be required. Third, the two-year duration of follow up time may be too short to identify clinically meaningful effects and to identify divergent trajectories for men and women. In addition, several other confounders such as the proportion of post-menopausal women taking HRT in this study, as well as the incidence of osteoporosis or other comorbidities common in MS and potentially exacerbated at menopause [33] could not currently be ascertained, which may have an effect on measured outcomes. Further studies are underway to assess this possibility.

In an era where unprecedented numbers of women with MS are entering the menopausal transition with relatively preserved clinical function, and for whom hormone modulation therapies, if effective, may present a different benefit: risk profile than in a healthy population [34,35], further inquiries into this topic are crucial, in order to confirm or contest these preliminary findings. Our analyses suggest that large sample sizes maybe required to complete the critical first step in this research avenue, which is to better understand the potential effect of menopause on disease activity.

## Abbreviations

BPF: Brain parenchymal fraction; CES-D: Center for epidemiological studies depression scale; CIS: Clinically isolated syndrome; CLIMB: Comprehensive longitudinal investigation of multiple sclerosis at the Brigham and women’s Hospital; CSF: Cerebrospinal fluid; EDSS: Expanded disability status scale; HRT: Hormone replacement therapy; MFIS: Modified fatigue impact scale; MRI: Magnetic resonance imaging; MS: Multiple sclerosis; NIH: National Institutes of Health; PPMS: Primary progressive multiple sclerosis; PRO: Patient-reported outcome; QOL: Quality of life; RRMS: Relapsing remitting multiple sclerosis; SF-36: Short-Form 36; SPMS: Secondary progressive multiple sclerosis; T2LV: T2 lesion volume.

## Competing interests

RB reports no disclosures. AM reports no disclosures. BCH has received research support from Merck Serono. MH has served as an advisor or consultant for Biogen Idec, EMD Serono, Teva Neuroscience, and has served as a speaker for Biogen Idec and EMD Serono. BIG has received research support from Merck Serono. SK has served as a consultant for Epivax and Novartis. CRGholds stock in Alnylam, Nestle, Novartis, Roche, and GSK. PLD has served as a consultant for Teva Neurosciences and Biogen-Idec. TC has served as a consultant for Biogen-Idec, Sanofi Aventis, Novartis, EMD-Serono, and Teva Neurosciences, and has received grant support from Merck-Serono for unrelated activities. The authors report no potential conflict of interest for this manuscript.

## Authors’ contributions

RB (rbove@partners.org) contributed to the design and drafting for intellectual contents of this manuscript. BH (bchealy@partners.org) and AM (amusallam@partners.org) contributed to the design, statistical analysis and drafting for intellectual contents of this manuscript. M. Houtchens (mhoutchens@partners.org), BG (bglanz@partners.org), SK(skhoury@partners.org) and CRG (cguttmann@partners.org) contributed to intellectual contents. PLDJ (pdejager@partners.org) and TC (tchitnis@rics.bwh.harvard.edu) contributed to design and drafting for intellectual contents. All Authors’ read and approved.

## Pre-publication history

The pre-publication history for this paper can be accessed here:

http://www.biomedcentral.com/1471-2377/13/73/prepub

## Supplementary Material

Additional file 1: Table S1 Cross-sectional and longitudinal patient-reported outcomes by sex for each age cohort (comparisons are provided between age cohorts and between sexes). Click here for file
