# Supplementary material for: No sex-specific difference in disease trajectory in multiple sclerosis patients before and after age 50
Source: BMC Neurol. 2013 Jul 3;13:73. doi: 10.1186/1471-2377-13-73 (PMC3707791; doi:10.1186/1471-2377-13-73)
Supplement: Additional file 1: Table S1 — Cross-sectional and longitudinal patient-reported outcomes by sex for each age cohort (comparisons are provided between age cohorts and between sexes). [file 1471-2377-13-73-S1.docx]

**Supplementary Table 1. Cross-sectional and longitudinal patient-reported outcomes by sex for each age cohort (comparisons are provided between age cohorts and between sexes).**

|  |  | **Females** | | | | **Males** | | | | **Males vs. Females** | | **Adjusted Interaction Analysis**  **Age cohort***  **Gender** |
| --- | --- | --- | --- | --- | --- | --- | --- | --- | --- | --- | --- | --- |
| **Cohort (years)** |  | **C1**  **(38-46)** | **C2**  **(54-62)** | **C1 vs. C2**  **p-value** | | **C1**  **(38-46)** | | **C2**  **(54-62)** | **C1 vs. C2**  **p-value** | **C1**  **p-value** | **C2**  **p-value** |  |
| **CROSS SECTIONAL DATA [mean, (SD)]** | | | | | | | | | | | | |
| CES-D |  | 29.5  (8.28) | 28.18 (8.24) | 0.26 | 30.94  (8.22) | | | 29.86  (8.53) | 0.62 | 0.3 | 0.43 | 0.93 |
| MFIS |  | 24.64 (16.27) | 28.52 (16.9) | 0.1 | 27.33 (20.23) | | | 28.24 (19.52) | 0.86 | 0.42 | 0.95 | 0.50 |
| SF-36 | PCS | 49.08 (8.99) | 45.34 (11.08) | **0.01** | 48.85  (9.14) | | | 46.44 (10.77) | 0.37 | 0.88 | 0.68 | 0.55 |
|  | MCS | 49.05 (10.02) | 50.03 (9.26) | 0.47 | 47.51  (9.12) | | | 48.11  (8.7) | 0.8 | 0.33 | 0.37 | 1 |
|  | Physical functioning | 50.83  (8.8) | 44.1 (12.48) | **<0.0001** | 48.11 (12.12) | | | 45.74 (12.47) | 0.46 | 0.15 | 0.59 | 0.11 |
|  | Role physical | 46.37 (11.78) | 42.71 (12.61) | **0.03** | 46.2  (12.25) | | | 44.13 (13.26) | 0.54 | 0.93 | 0.66 | 0.66 |
|  | Bodily pain | 51.64 (8.49) | 49.18 (11.72) | 0.07 | 50.66 (10.79) | | | 50.22  (10) | 0.87 | 0.57 | 0.68 | 0.43 |
|  | General health | 48.28 (9.13) | 50.11 (9.42) | 0.16 | 46.26 (10.63) | | | 46.15 (11.01) | 0.97 | 0.24 | 0.13 | 0.50 |
|  | Vitality | 48.15 (10.89) | 47.25 (10.7) | 0.55 | 46.22 (10.68) | | | 46.88  (9.69) | 0.8 | 0.28 | 0.88 | 0.61 |
|  | Social functioning | 50.2  (9.4) | 49.27 (10.15) | 0.5 | 48.14 (11.61) | | | 49.97 (10.68) | 0.52 | 0.27 | 0.79 | 0.30 |
|  | Role emotional | 47.74 (11.52) | 45.32 (12.74) | 0.16 | 46.2  (12.39) | | | 44.65 (13.18) | 0.64 | 0.45 | 0.83 | 0.67 |
|  | Mental health | 50.58 (8.79) | 51.61 (8.52) | 0.39 | 48.65  (9.1) | | | 48.83  (9.21) | 0.94 | 0.2 | 0.21 | 0.9 |
| **2-YEAR LONGITUDINAL DATA (slope of change)** | | | | | | | | | | | | |
| CES-D |  | 0.22 | 0.71 | 0.36 | -0.15 | | 0.06 | | 0.82 | 0.53 | 0.46 | 0.74 |
| MFIS |  | 0.17 | 1.05 | 0.27 | -0.48 | | 0.81 | | 0.37 | 0.55 | 0.85 | 0.86 |
| SF-36 | PCS | -0.06 | -0.35 | 0.5 | 0.20 | | 1.11 | | 0.26 | 0.63 | 0.05 | 0.16 |
|  | MCS | -0.36 | -1.30 | 0.17 | 0.26 | | -0.86 | | 0.33 | 0.42 | 0.63 | 0.99 |
|  | Physical functioning | -0.02 | -0.87 | **0.03** | -1.01 | | 0.77 | | 0.05 | 0.05 | **0.02** | **0.002** |
|  | Role physical | -0.51 | -1.84 | 0.09 | 0.37 | | 0.37 | | 0.99 | 0.34 | 0.13 | 0.35 |
|  | Bodily pain | 0.017 | 0.28 | 0.67 | -0.15 | | 1.61 | | 0.12 | 0.82 | 0.29 | 0.24 |
|  | General health | 0.36 | -0.35 | 0.13 | 0.89 | | -0.38 | | 0.19 | 0.38 | 0.98 | 0.61 |
|  | Vitality | -0.23 | -0.29 | 0.92 | 0.12 | | 0.50 | | 0.72 | 0.63 | 0.43 | 0.69 |
|  | Social functioning | -0.25 | -0.42 | 0.81 | -0.002 | | 0.29 | | 0.80 | 0.74 | 0.48 | 0.58 |
|  | Role emotional | -0.56 | -2.31 | 0.07 | -0.42 | | -1.05 | | 0.72 | 0.92 | 0.46 | 0.49 |
|  | Mental health | -0.04 | -0.54 | 0.36 | -0.13 | | -0.73 | | 0.53 | 0.93 | 0.91 | 0.96 |

LEGEND

^: N=294. All comparisons were made using an ANOVA for group comparisons and linear regression for adjusted analysis.
